# Targeting Deficiencies in the TLR5 Mediated Vaginal Response to Treat Female Recurrent Urinary Tract Infection

**DOI:** 10.1038/s41598-017-10445-4

**Published:** 2017-09-08

**Authors:** Ased S. M. Ali, Catherine Mowbray, Marcelo Lanz, Anna Stanton, Samantha Bowen, Claire L. Varley, Paul Hilton, Karen Brown, Wendy Robson, Jennifer Southgate, Phillip D. Aldridge, Alison Tyson-Capper, Soman Abraham, Robert S. Pickard, Judith Hall

**Affiliations:** 1 Institutes of Cell & Molecular Biosciences and Cellular Medicine, Newcastle University, Newcastle upon Tyne, UK; 2 Newcastle upon Tyne Hospitals NHS Trust, Newcastle upon Tyne, United Kingdom; 3 Duke University, Durham, NC USA; 4 Jack Birch Unit, University of York, York, UK; 5 Present Address: Mid Yorkshire Hospitals, Aberford Rd, Wakefield, UK

## Abstract

The identification of the host defence peptides as target effectors in the innate defence of the uro-genital tract creates new translational possibilities for immunomodulatory therapies, specifically vaginal therapies to treat women suffering from rUTI, particularly those carrying the TLR5_C1174T SNP. Urinary tract infections (UTIs) are a microbial disease reported worldwide. Women are particularly susceptible with many suffering debilitating recurrent (r) infections. Treatment is by antibiotics, but such therapy is linked to antibiotic resistance and re-infection. This study explored the innate protective mechanisms of the urogenital tract with the aim of boosting such defences therapeutically. Modelling UTIs *in vitro*, human vaginal and bladder epithelial cells were challenged with uropathogenic *Escherichia coli* (CFT073) and microbial PAMPs including flagellin, LPS and peptidoglycan. Flagellin functioning via the TLR5/NFκB pathway was identified as the key UPEC virulence factor causing a significant increase (P < 0.05) in the production of the host-defence peptide (HDP), BD2. BD2-depleted urine samples from bladder infected mice supported increased UPEC growth, strengthening the significance of the HDPs in protecting the urogenital tissues from infection. Clinically, vaginal-douche BD2 concentrations were reduced (p < 0.05) in women suffering rUTIs, compared to age-matched healthy controls with concentrations further decreased (p < 0.05) in a TLR5^392Stop^ SNP rUTI subgroup. Topical vaginal estrogen treatment increased (p < 0.001) BD2 concentrations in all women, including those carrying the SNP. These data identify therapeutic and antibiotic sparing roles for vaginal immunomodulatory agents that specifically target HDP induction, facilitate bacterial killing and disrupt the UPEC infection cycle.

## Introduction

Urinary tract infections (UTI), linked to *Escherichia coli* infecting the bladder are one of the most prevalent microbial diseases, accounting for over eight million health-care visits yearly in the United States^1^. Females are particularly susceptible^2^ and of those women affected 5%, rising to 13% in those aged over 60^3, 4^, will suffer from debilitating recurrent infections (rUTI). These infections not only impact on a person’s quality of life, but are also associated with escalating healthcare costs^1^. In a number of cases the recurrent infections can be linked to an abnormality of the urinary tract^5^, but a major characteristic of the disease is the poorly-defined relationship between the host genotype/phenotype and the main pathogen, uropathogenic *E*. *coli* (UPEC).

In healthy individuals, the lower urinary tract is maintained free of pathogens through the functioning of host defences that include the pH and ionic composition of urine, which in conjunction with the flushing action of urine are detrimental to bacterial adherence and growth. Evidence also suggests that these physical factors are supported by host innate, rather than adaptive, immune defence mechanisms with the high rates of UTI linked to the bladder’s inability to mount an antibody response^6^. Innate elements protecting the lower urinary tract include the activities of either constitutively or induced host defence molecules. Antimicrobial agents such as uromodulin facilitate bacterial removal by binding UPEC directly, while others, including lactoferrin and neutrophil gelatinase-associated lipocalin-2, function by sequestering iron to restrict bacterial survival and growth^7^. Small cationic peptides, synthesised by epithelia and neutrophils, and including the defensins, cathelicidin, and ribonucleases, are also part of the urinary tract innate defences^8^. These molecules, in addition to their intrinsic bacterial killing properties, fight infection through their immunomodulatory properties that promote increased cytokine production and neutrophil infiltration^9^. However, the actual roles of such peptides in protecting the urogenital tract from infection remain contentious. Studies involving mice deficient in the antimicrobial peptides Defb1 (analogous to human Beta-Defensin-1) and cathelin-related antimicrobial peptide (analogous to the cathelicidin hCAP-18) report conflicting results, with the absence of peptides associated with either increased, reduced or no differences in UTI susceptibility^10–13^.

Epithelial cells recognise potential pathogens through receptors including the Toll-like receptors (TLRs). Once activated, the TLRs induce a rapid response that results in microbial killing either directly through the synthesis and activities of antimicrobial agents or indirectly through chemokines, and the attraction of neutrophils and macrophages. Studies focussed on UPEC infection of the murine urinary tract suggest that TLR4 activation, linked to the detection of microbial lipopolysaccharide and/or FimH, the adhesin portion of the Type 1 fimbriae that secures UPEC attachment to urothelial cells, can trigger host innate antimicrobial defences^14^. Following urothelial cell invasion, TLR4 is further implicated in the expulsion of UPEC and reinfection of the urinary tract, via an exocytic pathway linked to cellular cAMP^15^. However, *in vitro* data using proliferating and differentiated normal urothelial cells favours TLR5 signalling mechanisms predominating in the human bladder^16^. In support, murine studies have also demonstrated bacterial motility and TLR5 to be a key factors in UPEC pathogenicity, with wild-type flagellated UPEC shown to out-compete non-flagellated *fliC* mutants and maximum expression of flagellar genes coinciding with UPEC ascent into the ureters and kidneys^17, 18^. Clinically, patient susceptibility to UTI links to polymorphisms of both receptor genes with a TLR4_A896G polymorphism related to rUTI protection, but a TLR5_C1174T SNP linked to an increased susceptibility to repeated infections^19, 20^.

Current management strategies for rUTI sufferers are generally prophylactic involving long-term low dose antibiotics. In reality such strategies provide limited long-term benefit and encourage bacterial resistance that further complicates patient management. The treatment also conflicts with the current public health challenges of managing and reducing antibiotic overuse^21, 22^. Increasing concerns relating to the overuse of antibiotics has stimulated interest in alternative therapies including vaccine development and identification of agents able to boost endogenous innate defences of the urogenital tract^8^. In females the initiating event of an UTI involves colonisation of the vaginal mucosa by UPEC originating, it is presumed, from the gut microbiota^23^, with UTI caused by the subsequent urethral migration and attachment of these bacteria to the urothelium^24^.

We show here that not only does deficiency in the vaginal host antimicrobial defences link to recurrent UTIs, but also that vaginal innate defences can be enhanced through topical immune boosting agents. We propose that such agents can be utilised to disrupt the UPEC infection cycle and hence offer an effective antibiotic-sparing approach to the treatment of rUTIs.

## Results

### *E*. *coli* and Flagellin induce a TLR5-Mediated BD2 Response in Urogenital Epithelia

**Figure 1:**
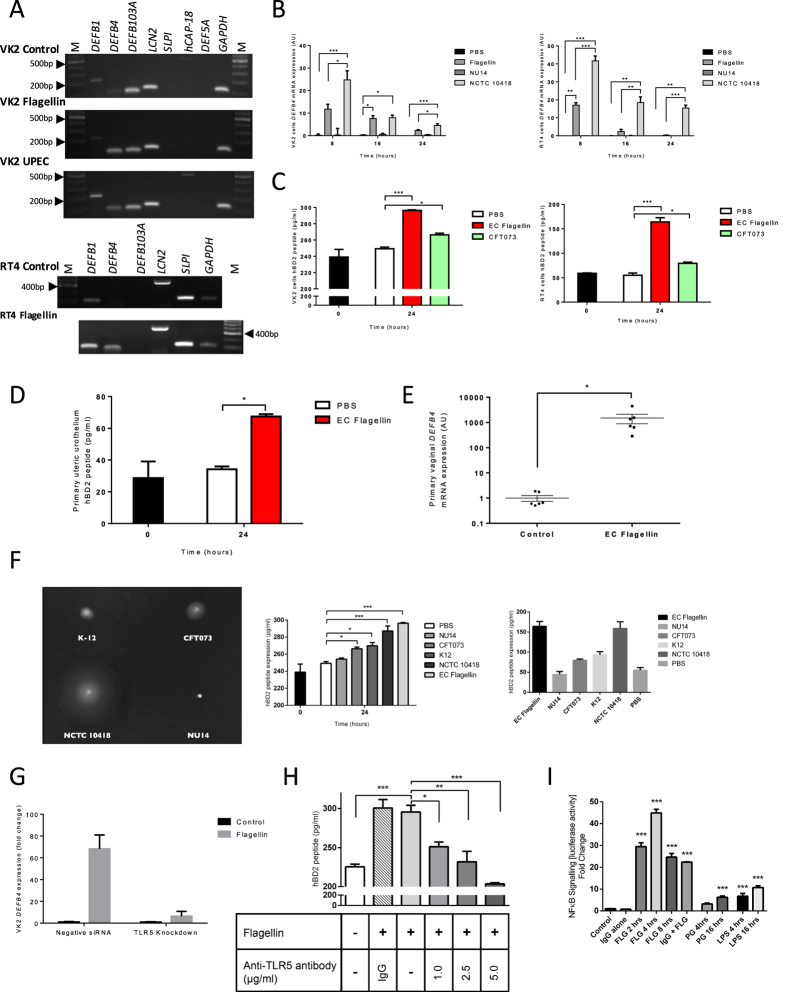
VK2 E6/E7 and RT4 *DEFB4* and BD2 responses to UPEC and flagellin challenges. End point PCR panels of host defence peptide gene expression in VK2 E6/E7 and RT4 cells following 24 hr challenge with PBS (control), flagellin (250 ng/ml) or UPEC (10^5^) (**A**). Full length gels are shown in Supplementary File 1 (**S1A**–**D**). *DEFB4* transcript expression in VK2 E6/E7 and RT4 cells following challenges with flagellin (250 ng/ml) and heat-killed flagellated (NCTC10418) or non-flagellated (NU14) *E*. *coli* (10^5^) (N = 3, n = 9) (**B**). BD2 concentrations measured in VK2 E6/E7 and RT4 cell media following 24 hour challenges with flagellin (250 ng/ml) and heat-killed CFT073 (10^5^) (N = 3, n = 6) (**C**). BD2 peptide concentrations of primary ureteric urothelial cells challenged with *E*.*coli* flagellin (250 ng/ml) for 24 hours (N = 3, n = 6) (**D**). *DEFB4* transcript expression, presented as relative expression, of primary vaginal epithelial cells challenged with *E*.*coli* flagellin (50 ng/ml) for 24 hours (N = 6) (**E**). *E*.*coli* swarming motility on motility agar media; BD2 peptide concentrations measured in VK2 E6/E7 and RT4 cell media following 24 hour challenges with flagellin (250 ng/ml) and heat-killed bacteria (10^5^) (N = 3, n = 6) (**F**). *DEFB4* transcript expression of VK2 E6/E7 cells transfected with either negative siRNA or TLR5 siRNA and challenged for 24 hours with flagellin (50ng/ml) (N = 3) (**G**). BD2 peptide concentrations of VK2 E6/E7 cells incubated with TLR5 blocking antibody (1–5 ug/ml) or IgG (5 ug/ml) and challenged 24 hours with flagellin (250 ng/ml) (N = 3) (**H**). Fold change in NFĸB-luciferase activity in RT4 cells challenged with 250 ng/ml flagellin (FLG) for 2–8 hours, peptidoglycan (PG) (10 μg/ml) for up to 16 hours and Lipopolysaccharide (LPS) (10 μg/ml) for up to 16 hours (N = 3, n = 6 (FLG), n = 9 (PG & LPS)) (**I**). All data presented as mean ± SEM, *P < 0.05, **P < 0.01, ***P < 0.001.

Immortalised vaginal (VK2 E6/E7) and bladder cancer (RT4) cells modelling the urogenital epithelial tissues were challenged with a heat-inactivated suspension of the motile flagellated UPEC strain CFT073 and *E*.*coli* flagellin. Expression of genes *DEFB1*, *DEFB4*, *DEFB103A*, *LCN2*, *SLPI*, *hCAP-18* and *DEF5A* encoding the host defence molecules BD1, BD2, BD3, Lipocalin2, secretory leukocyte protease inhibitor (SLPI), cathelicidin and HD5 were assessed using end-point PCR. While these data suggested *DEFB1* and *DEFB4* gene expression to be upregulated (Fig. 1A and Supplementary S1a–d), qRT-PCR analyses identified only a significant increase in *DEFB4* gene expression (Figs 1B and S2) with these observations accompanied by a significant increase in BD2 peptide following CFT073 (P < 0.05) and flagellin (P < 0.001) challenge (Fig. 1C). These data were substantiated using primary human ureteric urothelial and vaginal epithelial cells (Fig. 1D and E). Hence we selected BD2, an inducible bacterial killing molecule and immunomodulatory agent with reported potency in inhibiting UTI progression^25^ as our key target in exploring and devising strategies to boost the UT innate defences.

Challenging VK2 E6/E7 and RT4 cells with flagellated (motile) and non-flagellated (non-motile) *E. coli* indicated that the signalling mechanism controlling BD2 production in the urogenital tissues was TLR5 mediated and linked to flagellin (Fig. 1F). This was further indicated by a reduction in *DEFB4* transcript expression in flagellin challenged vaginal VK2 E6/E7 cells following siRNA silencing of TLR5 gene expression (Fig. 1G). In support decreased BD2 concentrations were also detected in the media bathing the vaginal cells similarly challenged with flagellin, but in which TLR5 had been blocked using antibody (Fig. 1H). The use of RT4 bladder cells engineered to contain a NFκB-luciferase reporter also indicated the NFκB signalling pathway to be involved with a four hour flagellin challenge linked to a significant 44.8 fold increase in luciferase activity (P < 0.001) (Fig. 1I). In comparison only slight induction of NFκB activity was observed at four hours following either peptidoglycan (3.1 fold) or lipopolysaccharide (LPS) challenges (6.7 fold) with maximal responses at 16 hours of 6.2 and 10.7 fold respectively (Fig. 1I). However, LPS challenges of up to 24 hours *in vitro* did not induce *DEFB4* expression (Fig. S3). These data identified TLR5/NFκB/BD2 as a key signalling pathway in the innate response of the urogenital epithelia to a flagellated UPEC infection.

### Deficiency of BD2 Orthologue (DefB4) in Mice Increases Susceptibility to UTI

**Figure 2:**
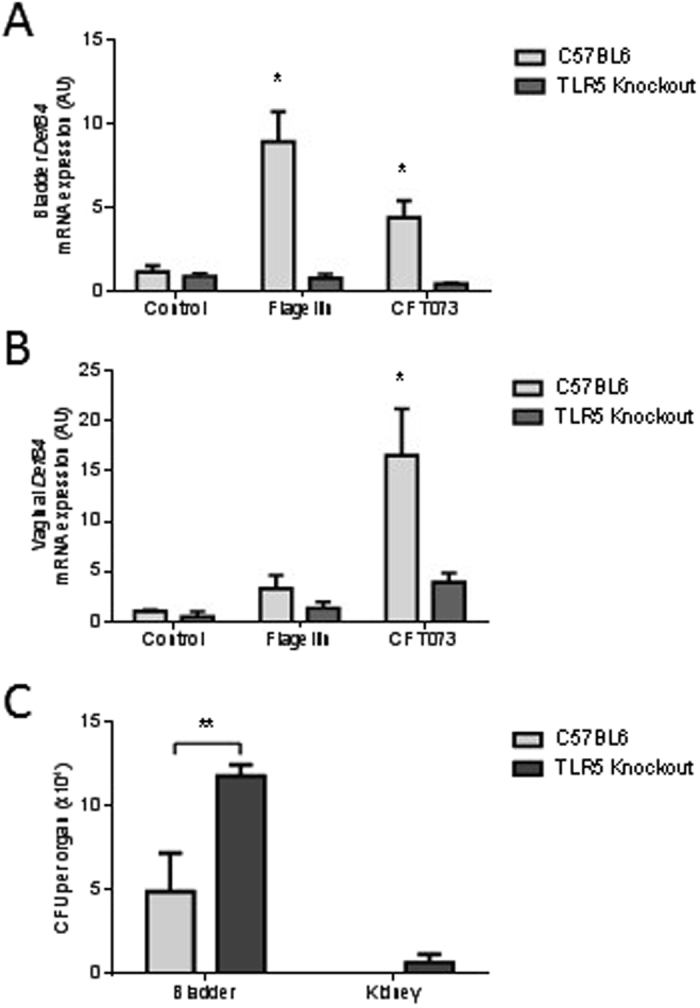
*DefB4* expression in mouse urogenital tissues following UPEC and flagellin challenge. *DefB4* mRNA expression in wild type C57BL6 and *Tlr5* ^−/−^ mice bladders following 6 h challenge with either flagellin (5 µg) or CFT073 (10^8^) (N = 3) (**A**). Vaginal *DefB4* mRNA expression in wild type C57BL6 and *Tlr5* ^−/−^ mice following 6 h challenge with either flagellin (5 µg) or CFT073 (10^8^) (N = 3) (**B**). *E*. *coli* colonisation of the bladders and kidneys of wild type C57BL6 and *Tlr5* ^−/−^ mice following 6 h CFT073 bladder challenge (N = 3) (**C**). All data presented as mean ± SEM, *P < 0.05, **P < 0.01.

To verify this pathway and the significance of host defence peptides including BD2 *in vivo*, C57BL/6 J WT and *Tlr5* ^−/−^ knockout mice bladders were inoculated with either buffer (PBS), *E*.*coli* derived flagellin (5 µg) or CFT073 (10^8^) and the murine bladder and vaginal tissues examined for *DefB4* gene expression. The *DefB4* gene is the mouse orthologue of the human *DEFB4* gene^26^. C57BL/6 J WT mice showed a significant increase in bladder *DefB4* transcript expression (P < 0.05) in response to the flagellin challenge (Fig. 2A), while significant changes in both bladder and vaginal expression (P < 0.05) were associated with the bacterial (CFT073) infection (Fig. 2A,B). No renal *DefB4* transcript expression was identified (data not shown) and no bacteria were recovered from the kidneys (Fig. 2C).

The bladder and vaginal tissues of similarly challenged *Tlr5* ^−/−^ mice showed no significant induction of *DefB4* transcript expression (Fig. 2A,B). At six hours post challenge the bladder bacterial counts of these mice were also significantly higher than those recorded in the C57BL/6J WT mice (Fig. 2C). Additionally bacteria were recovered from the kidneys of the *Tlr5* ^−/−^ mice indicating the increased susceptibility of their urinary tracts to an ascending UTI (Fig. 2C). These *in vivo* data supported the significance of the urogenital epithelial TLR5/BD2 host defence peptide (HDP) signalling response in the immediate protection of the lower and upper urinary tract from UPEC infection.

### Biological Relevance of BD2 in the Innate Response of the Uro-genital Tract

**Figure 3:**
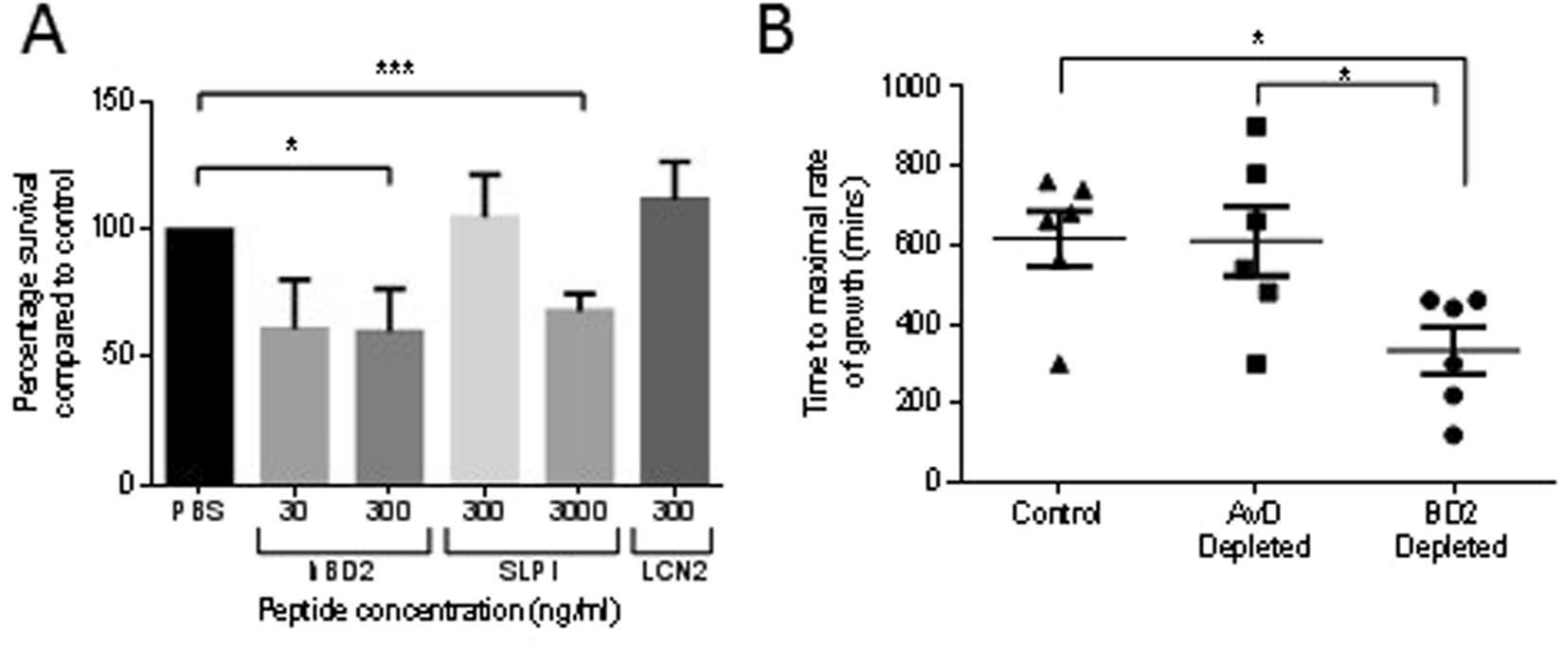
Effects of host defence peptides on CFT073 growth. Time-kill data showing CFT073 survival following two hour incubation with host defence peptides BD2 (30 and 300 ng/ml: 7.5 nM and 75 nM), LCN2 (300 ng/ml: 13 nm)) and SLPI (300 and 3000 ng/ml: 25 and 250 nM) (N = 4). Bacterial growth values >100% link to peptide concentrations not associated with bacterial killing suggesting amino acids associated with peptide degradation, either natural or bacterial protease activity, are used by the bacteria as a growth source (**A**). CFT073 (10^4^) growth times in wild type mice urines depleted of DefB4 (controls: no depletion and Avian defensin antibody-specific depletion) (**B**). All data presented as mean ± SEM, *P < 0.05, ***P < 0.001.

To address the biological relevance of host defence peptides in the innate defence of the urogenital tissues the antimicrobial potencies of an array of agents including BD2, LCN and SLPI (7.5 to 250 nM) were compared using a time-kill assay approach and CFT073 as the target bacterium. Using peptide concentrations mimicking those at the epithelial surface^27, 28^ these assay data suggested the induced BD2 peptide to possess significant antimicrobial potency against the CFT073 strain (Fig. 3A). Immuno-depletion of BD2 from the wild-type mouse urine samples collected following challenge with CFT073 and flagellin was also associated with increased CFT073 growth (Fig. 3B). These data provided direct evidence that a urogenital deficiency of host defence peptide links to the increased risk of UTI.

### Women with rUTI and TLR5_C1174T SNP show Reduced BD2

**Figure 4:**
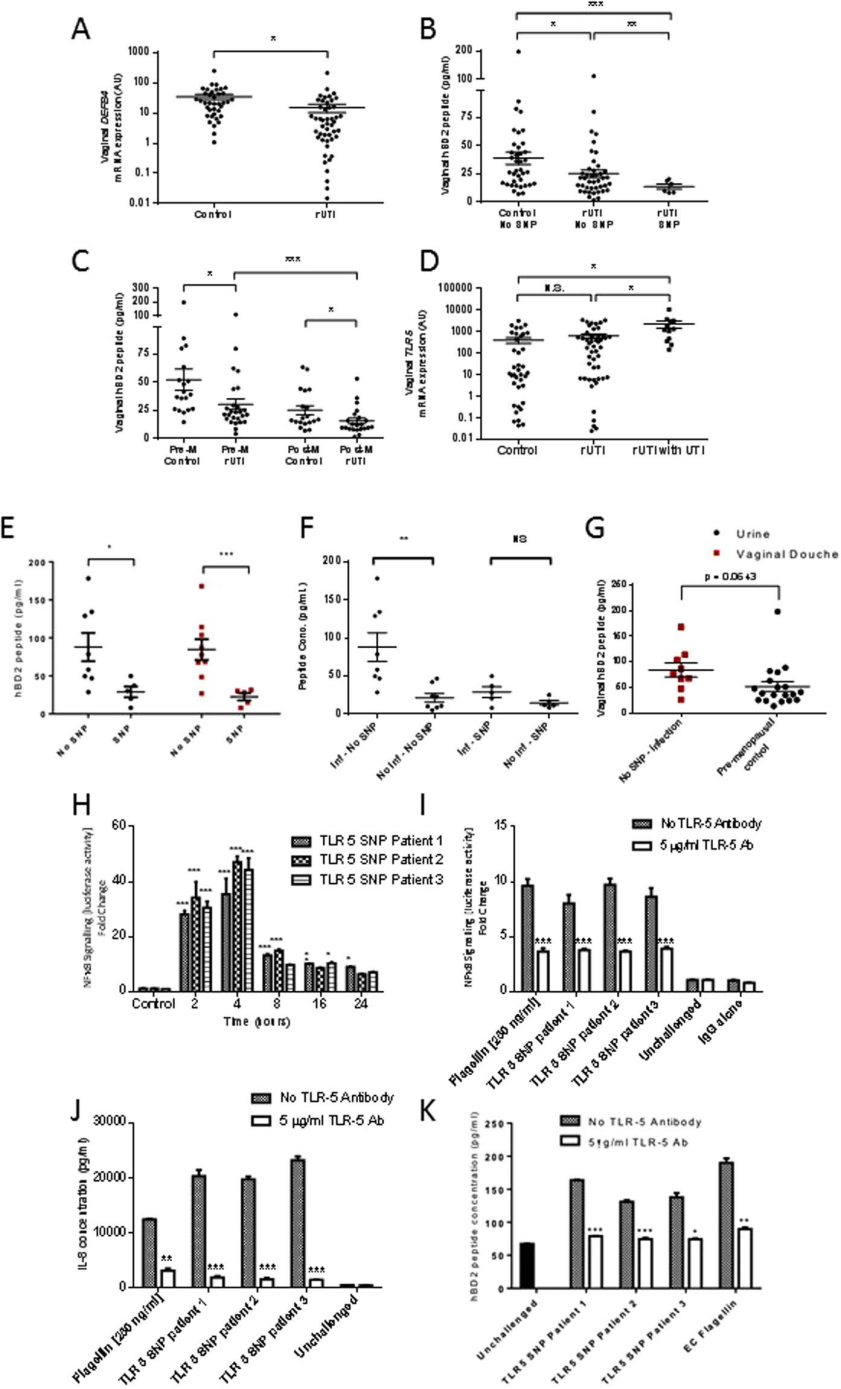
*DEFB4* and *TLR5* mRNA expression, and BD2 responses in control and rUTI patient samples. *DEFB4* mRNA expression, presented as relative expression (**A**) and BD2 peptide concentrations (**B**) in vaginal biopsies from control (N = 38) and rUTI patients (N = 48). Vaginal douche data stratified to show BD2 peptide concentrations of premenopausal (Pre-M) control subjects (N = 19), premenopausal rUTI patients (N = 26), postmenopausal (Post-M) control subjects (N = 19) and postmenopausal rUTI patients (N = 22) (**C**). *TLR5* mRNA expression, presented as relative expression, in vaginal biopsies from control subjects, patients with rUTI and rUTI at time of an active infection (**D**). Urine BD2 concentrations of the rUTI No SNP (N = 8) and TLR5^392S*top*^ SNP patients (N = 5) and vaginal douche BD2 peptide concentrations N = 9 (No SNP) and N = 5 (SNP) during periods of active infection (**E**). Matched urine BD2 concentrations of rUTI non SNP (N = 8) and TLR5^392S*top*^ SNP patients (N = 5) analysed during periods of infection and non-infection (**F**). Vaginal douche BD2 peptide concentrations of no SNP rUTI patients measured during an acute infection alongside vaginal douche BD2 concentrations of the non-infected pre-menopausal control subjects (**G**). NFĸB-luciferase activity (fold change) in RT4 cells following 2 to 24 hour challenges with heat killed UPEC (5 × 10^4^) isolated from the urines of three rUTI SNP patients during an acute infection (**H**). NFĸB-luciferase activity (fold change) in RT4 cells incubated in presence or absence of TLR5 blocking antibody (5 ug/ml) or IgG (5 ug/ml) following 24 hour challenge with flagellin (250 ng/ml) or heat killed UPEC isolated from each of the three SNP patients (**I**). IL-8 concentrations (pg/ml) in media of RT4 cells incubated in presence or absence of TLR5 blocking antibody (5 ug/ml) and challenged for 24 hours with flagellin (250 ng/ml) and bacteria isolated from each of the three SNP patients (**J**). BD2 concentrations (pg/ml) in media of RT4 bladder cells incubated in presence or absence of TLR5 blocking antibody (5 ug/ml) and challenged for 24 hours with either flagellin (250 ng/ml) or bacteria isolated from each of the three SNP patients (**K**). N = 2, n = 6 and all data presented as mean ± SEM, *P < 0.05, **P < 0.01, ***P < 0.001.

To examine the clinical significance of host defence peptides in defending against rUTIs we focused on *DEF4B* expression and BD2 synthesis in vaginal biopsies, vaginal secretions (douche) and urine samples from 86 women. Forty-eight women had a history of rUTIs, including 14 with a symptomatic *E*.*coli* infection at time of sampling and 38 were age-matched controls. Analyses of the vaginal biopsies indicated that those women suffering rUTIs showed significantly lower *DEF4B* transcript expression (p < 0.05) compared to their respective controls (Fig. 4A). The vaginal douche measurements also supported these data with the mean BD2 concentration being significantly reduced (P < 0.05) in the rUTI cohort (25.3 ± 3.2 pg/ml) compared to the control group (38.9 ± 5.5 pg/ml) (Fig. 4B). Stratification of these vaginal douche data also showed that the mean BD2 concentrations of the pre and postmenopausal rUTI cohorts were significantly decreased (p < 0.05) compared to their respective controls (Fig. 4C). Furthermore, the mean BD2 concentration of the postmenopausal rUTI group was significantly decreased (p < 0.001) compared to that of the premenopausal rUTI cohort.

TLR5 gene expression data suggested no significant differences between vaginal expression in the control and rUTI cohorts, and expression was elevated significantly (P < 0.05) during active infections (Fig. 4D). These data suggested that the observed reductions in BD2 were related to cellular signalling events linked to and/or downstream of TLR5. To explore this further we sequenced the TLR5 genes of the patient and control cohorts. Sequencing identified a sub-group of six women (7%), all contained within the rUTI group, who carried the TLR5_C1174T single nucleotide polymorphism (SNP). Also known as TLR5^392S*top*^, this polymorphism is a heterozygous variant present in 5–10% of the population encoding a stop-codon, which is reported to increase the susceptibility of those affected to flagellated infections^29, 30^. Vaginal douche measurements also supported HDP concentrations being significantly lower (P < 0.01) in this group compared to the no SNP rUTI cohort (Fig. 4B).

Of the fourteen women with an active UTI during our sample collections, nine carried the wild-type TLR5 gene and five the TLR5^392S*top*^ SNP. Analyses of urines and vaginal washings of these women during their active infections showed that the SNP cohort had significantly lower mean BD2 concentrations in both urine (P < 0.05) and vaginal douche (P < 0.001) samples compared to those of the no SNP group (Fig. 4E). Furthermore, analysing the urine BD2 concentrations of the TLR5^392S*top*^ SNP cohort during periods of infection and quiescence showed no significant differences (Fig. 4F), confirming the inability of the urogenital tissues of these SNP patients to respond to infection. In contrast the urine BD2 concentrations of the no SNP rUTI patients were elevated (P < 0.01) in response to infection (Fig. 4F). However, further analyses showed that the BD2 concentrations of the no SNP rUTI patients measured during an active UTI were actually very similar to those of the control pre-menopausal group (Fig. 4G). These data suggested that the vaginal BD2 responses in the no SNP rUTI patients were considerably compromised, which increased their susceptibility to recurrent infections. The diminished host BD2 response of the no SNP rUTI group implicated host signalling defects linked to and/or downstream of TLR5.

To demonstrate that the rUTIs suffered by the SNP patients were due to host-susceptibility rather than bacterial virulence factors, *E*.*coli* were isolated from the urine samples of three of the SNP patients and used to challenge the stably transfected RT4-NFκB luciferase reporter cells. Significant increases in NFκB-reporter activity comparable to flagellin (250 ng/ml) were recorded following challenges with each of the UPEC strains (Fig. 4H). These data verified that the genetic TLR5^392S*top*^ polymorphism played a key role in predisposing these women to repeated UTIs. Modelling the polymorphism experimentally by TLR5 antibody blocking of the RT4-NFκB luciferase reporter cells, prior to bacterial challenges, reduced NFκB reporter activity (Fig. 4I) and diminished the epithelial effector IL-8 and BD2 responses (Fig. 4J,K). Consistent with the bladder response (Fig. 4K) and patient data a reduction in BD2 was observed in similarly challenged vaginal cells (Fig. S4). These data provided further evidence that in the urogenital tract the epithelial host TLR5-NFκB-HDP signalling pathway functions in helping to protect from UTIs and if compromised, the loss of such defences, can expose the tissues to infection.

### Deficient BD2 Responses can be Enhanced Therapeutically *In Vitro* and Clinically

**Figure 5:**
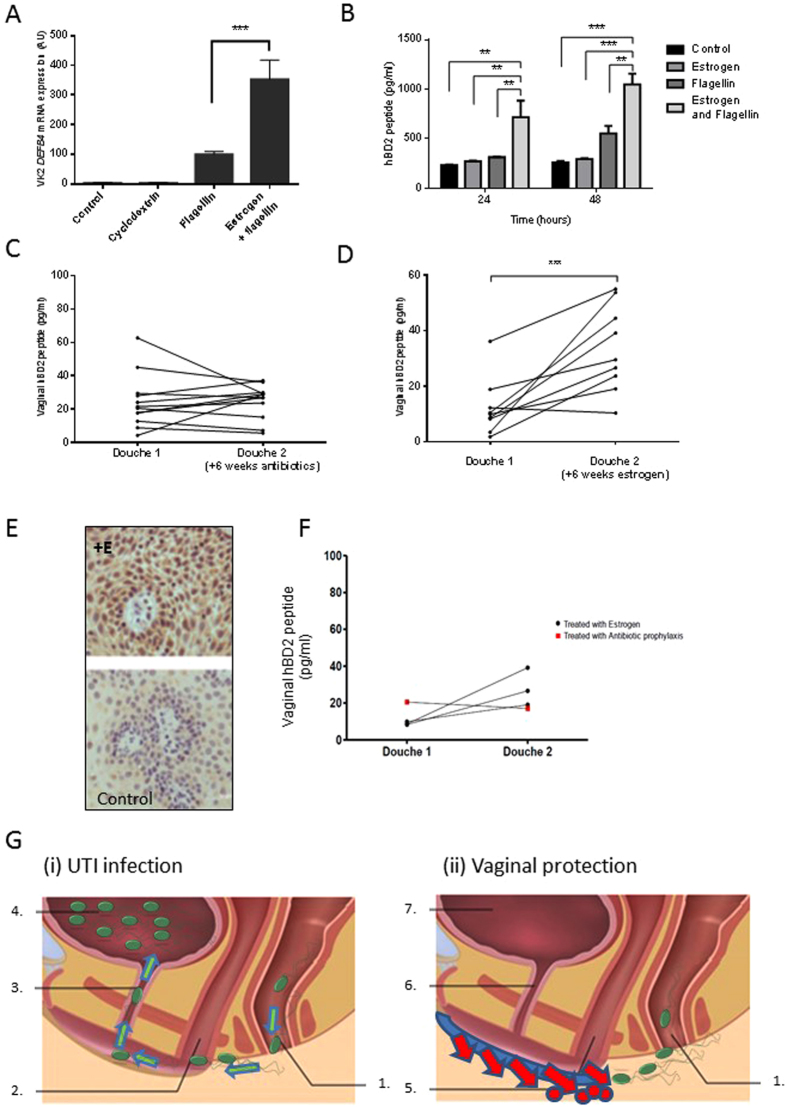
Effects of estrogen treatment on BD2 responses *in vitro* and rUTI patients. VK2 E6/E7 cell *DEFB4* mRNA expression following estrogen (4 nM) seven day pretreatment and 24 h challenge with either cyclodextrin (15 nM), flagellin (50 ng/ml), or flagellin (50 ng/ml) and estrogen (4 nM) N = 3 (**A**). BD2 peptide concentrations measured in the VK2 E6/E7 cell media following estrogen (4 nM) seven day pretreatment and either 24 h or 48 h challenge with either cyclodextrin (15 nM), estrogen (4 nM), flagellin (50 ng/ml) or flagellin and estrogen (N = 3) (**B**). Vaginal douche BD2 peptide concentrations of rUTI patients before and after 6–8 weeks of treatment with antibiotic prophylaxis and/or advice (N = 12) (**C**), or 6 to 8 weeks treatment with vaginal estrogen (Vagifem, 10 mcg, twice weekly) (N = 9) (**D**). Staining of vaginal tissue from estrogen treated ( + E) and control subjects for immunoreactive BD2 (**E**). Vaginal douche BD2 peptide concentrations of rUTI TLR5 SNP patients before and after treatment (6 to 8 weeks) with vaginal estrogen (N = 3) or antibiotic prophylaxis (N = 1) (**F**). All data presented as mean ± SEM, **P < 0.01, ***P < 0.001. Schematic illustrating (i) how an inherited or acquired reduction in the vaginal host innate defences can facilitate an UPEC infection of the urogenital tract and (ii) how vaginal treatments enhancing the host innate defences can help prevent this (**G**). **1**. Uropathogenic *E*.*coli* (UPEC)  originating from bowel migrate from rectum towards vagina; **2**. Inherited or acquired reduction in innate defences including HDP and chemokines facilitates increased vaginal and peri-urethral colonisation by UPEC; **3**. Urethral ascent of UPEC from peri-urethral and vaginal regions to bladder; **4**. Bladder infection; **5**. Vaginal topical agent stimulates host innate defences and UPEC destroyed preventing vaginal colonisation; **6/7**. Urethra and bladder protected from UPEC infection.

For all rUTI patients, including those carrying the TLR5 SNP, topical agents that boost the urogenital defences, including BD2 synthesis, may provide additional and/or alternative therapies to antibiotics. Vaginal estrogen supplements can be used to reduce UTI risk in post-menopausal women suffering rUTIs, but the mechanisms of action are not fully understood^31–33^. When VK2 E6/E7 vaginal cells were supplemented with 4 nM estrogen for 7 days and then challenged with flagellin (50 ng/ml) *DEF4B* transcript expression and BD2 synthesis were significantly (P < 0.01) potentiated (Fig. 5A,B). These *in vitro* data suggest that the steroid hormone, in addition to its roles in reproduction, functions in augmenting the innate immune defences of the urogenital tract. When examined clinically, the vaginal BD2 concentrations of post-menopausal women suffering rUTIs, but prescribed topical vaginal estrogen (Vagifem 10 mcg twice weekly), for a minimum of six weeks were significantly increased (P < 0.001), compared to those treated using other therapies including antibiotic prophylaxis and/or advice (Fig. 5C,D). These vaginal douche data were supported by vaginal biopsies taken from estrogen-treated women that showed immunoreactivity for BD2 (Fig. 5E). Together these data provide compelling evidence that estrogen can enhance the host vaginal epithelial defences through the increased synthesis of host defence molecules and help protect from rUTIs. The fact that the BD2 vaginal douche concentrations of the rUTI TLR5 SNP patients were augmented in response to topical vaginal estrogen treatment (Fig. 5F), lends support to the use of immune-boosting vaginal therapies to help treat rUTIs.

We propose therefore that the TLR5-NFκB-host defence effector pathway is a significant innate defence mechanism helping to protect the urogenital tract against rUTI. We propose that for women carrying the TLR5 SNP and hence genetically predisposed to rUTI, as well as post-menopausal women, this pathway be exploited clinically. We argue that the use of topical immune modulating therapies that activate and/or enhance the vaginal innate defences will help prevent bacterial colonisation and ascension into the urinary tract (Fig. 5G).

## Discussion

UTI is one of the most prevalent microbial diseases worldwide. It is characterised by high recurrence rates amongst sufferers and by the marked, and increasing multi-drug resistance profiles of uropathogenic bacteria, a consequence of the mainstay therapy being limited to repeated or prophylactic antibiotic treatments^34, 35^. Key challenges to science and medicine are to reduce recurrence rates and improve patient outcomes through new therapeutic options that curtail antibiotic usage.

To address this our studies focussed on both bacterial virulence factors and the host urogenital defences. In support of previous reports^16, 18^ our *in vitro* and murine data indicated that flagellated *E*. *coli* through TLR5 activation play a key role in the pathology of UTI. These data were further strengthened by our clinical findings, which showed that all patients carrying the TLR5_C1174T SNP, encoding a stop mutation linked to increased susceptibility to flagellated bacteria^20^, suffered from recurrent UTIs. The fact that the *in vitro*, murine and clinical study data were all characterised by the reduced production of host defence molecules, including bacterial killing peptides and chemokines, provided a mechanistic explanation to link a TLR5 deficiency to repeated infections of the bladder. Furthermore this immunodeficiency impacted similarly on the TLR5 signalling pathway of the vaginal tissues. In view of this we propose that the resultant reduction in innate effector molecules facilitates the repeated colonisation of the vaginal mucosa by gut *E*. *coli*, which drives the persistent bladder infections. This mechanism also explains why antibiotic therapy can never cure women carrying the TLR5^392S*top*^ SNP and in fact why repeated treatments only exacerbate the condition through selecting for antibiotic resistance. Hence as a first step we propose that women suffering uncomplicated rUTI should be tested for the SNP to further inform their clinical management. However, herein lies the next problem as there are few clinical alternatives to treating rUTIs other than advice and antibiotics.

Topical estrogen treatment is an option in reducing UPEC colonisation of the vaginal tissues and helping to protect against repeated infections. Our clinical data, which showed elevated vaginal BD2 concentrations in patients prescribed estrogen treatment corroborated a previous report where estrogen therapy enhanced the bladder innate response and strengthened the integrity of the urothelium^32^. Our premise that estrogen functions synergistically to boost the urogenital innate defences was further supported by our observation of a significant reduction in the urine and vaginal douche BD2 concentrations of postmenopausal patients suffering rUTI, but carrying wild-type TLR5. The report that vaginal estrogen is also associated with increased *Lactobacilli* growth, which impacts on the survivability of uropathogens^36^, further supports its use as a rUTI therapy. Yet, despite its potential, vaginal estrogen is clinically acceptable only in post-menopausal women and due to concerns including endometrial hyperplasis^37^ is only recommended for limited periods. However, other potential agents for therapeutic consideration-include hyaluronic acid and chondroitin sulphate, whose intravesical use have been linked to decreased UTI recurrence and *in vitro* to host defence peptide stimulation, including increased defensin synthesis^38–40^.

Our vaginal therapy option to help treat rUTI sufferers carrying the TLR5 SNP (Fig. 5G) is based on the premise that recurrence links to infections that are propagated through bacteria originating in the gut-faecal material ascending the urethra into the bladder^23, 41, 42^. However, recurrent infections can also be explained using the intracellular bacterial community/quiescent intracellular reservoir (IBC/QIR) model established through studies in mice^43^. This model relies on UPEC ascending the urethra, invading the bladder epithelium and forming either IBCs that contain metabolically-active bacteria capable of infecting adjacent cells or non-replicating quiescent intracellular reservoirs. Following a stimulus, possibly months after the initial infection and potentially involving urothelial turnover involving actin rearrangements, these latent bacteria emerge to initiate a new acute infection. However, while the IBC/QIR model has been demonstrated in multiple mouse backgrounds^44^ there is limited evidence to support its functioning in humans^45, 46^. Furthermore, the infection model does not fit well with data that links vaginal treatments to reduced UTI incidence^31, 33^.

The answer to developing rUTI therapies applicable for all groups of women lies in understanding the disease pathology in relation to both the host response and bacterial virulence. To date, studies investigating UTIs and potential new UTI treatments have focussed largely on the virulence factors used by uropathogens at the primary site of infection, the bladder^43^. While such information is not generally taken into account in clinical decisions involving antibiotics, it has informed potential treatment strategies including vaccination. However, the use of vaccines to treat rUTIs remains tentative with whole pili immunogens proving ineffective and other methods focussed on UPEC toxins, siderophores and the FimC-FimH complex, either providing no protection or reducing, but not totally inhibiting bladder colonisation^47, 48^. Oral therapeutics called mannosides, which function as FimH anatagonists and reduce bacterial attachment at the bladder tissues show strong potential with the efficacy of a new class, the *C*-mannosides, demonstrated *in vivo* using animal models of UTI^49, 50^. Recently the FimH antagonist M4284 has been shown to reduce UPEC colonisation of the mouse gut without impacting on the gut microbiota, and hence if developed therapeutically, could reduce UTIs and rUTIs^51^. Deliberately establishing asymptomatic bacteruria in UTI-prone patients through the use of non-adhering *E*.*coli*, has also been reported to be relatively successful in reducing UTI episodes^52^. However, from our knowledge and understanding of the host innate defences we also advocate the development and use of topical vaginal treatments that function, either by mimicking the action of estrogen or through novel signalling pathways, to boost the endogenous innate defences of the vaginal tissues and reduce vaginal *E*.*coli* colonisation (Fig. 5G). We propose that such treatments, by interrupting the UPEC infection cycle, will offer an additional, but simple antibiotic-sparing therapeutic approach to the treatment of patients suffering rUTIs including those carrying the TLR5^392S*top*^ SNP.

## Methods

### Cell Culture

The RT4 urothelial cell line (ATCC HTB-2)^53^ was maintained without antibiotics in 25 mM HEPES (4-[2-hydroxyethyl]-1-piperazineethanesulfonic acid) modified RPMI 1640 medium supplemented with 2 mM glutamine and 10% fetal bovine serum (Sigma, Dorset, UK). VK2 E6/E7 cells (ATCC CRL-2616)^54^ were maintained without antibiotics in keratinocyte serum-free medium (GIBCO, Paisley, UK) containing 0.4 mM calcium with 0.1 ng/ml human recombinant Epidermal Growth Factor (EGF) and 0.05 mg/ml bovine pituitary extract supplements. Normal human urothelial cells were isolated, expanded and maintained in culture as previously described^55^.

### Cell Challenge Studies

RT4 and VK2 E6/E7 cells were seeded at 10^4^ cells/well into 30 mm, six well plates and cultured to confluency at 37 °C in 5% CO_2_. Prior to challenge, cell monolayers were washed in phosphate buffered saline (PBS) and incubated for 24 h in fresh medium.

NU14^56^, NCTC 10418 (ATCC 10536), K12^57^ and CFT073^58^ bacterial cultures were tested for motility as described previously^59^. Bacterial cultures were prepared for use in cell challenge experiments as follows: Fifty µl of a 5 ml log phase culture was used to inoculate a further 5 ml of medium and cultured again to log-phase (3 h). Twenty μl of this culture was re-suspended in 980 μl PBS to give a working suspension and 20 μl of this (approximately 5 × 10^4^ colony forming units (CFU)) used to inoculate each well of eukaryote cells for the time required. Dead bacteria were produced by incubating live bacterial stock solution for 30 minutes at 65 °C and confirmed by overnight agar plating. For bacterial component challenges, each well of eukaryote cells was inoculated with 20 μL of either LPS, Peptidoglycan (Invivogen, San Diego, California, USA) or *E*. *coli* flagellin^60^. For BD2 enhancement studies cells were supplemented with 4 nM cyclodextrin encapsulated 17β-oestradiol dissolved in water (Sigma) or 15 nM (2-hydroxypropyl)-β-cyclodextrin for up to seven days prior to challenge. Once the cells reached 80% confluency the cell culture medium was removed, the cells were washed with PBS and incubated a further 24 hours in medium supplemented with 15 nM cyclodextrin only. At each appropriate challenge time points the medium was removed from the wells and stored at −20 °C for ELISA and the cells subjected to RNA extraction.

The time-kill antimicrobial assays using recombinant peptides, BD2 (Preprotech), LCN2 (Biovision), and SLPI (RD systems) were adapted from the technique used previously^61^.

### NFκB Reporter Measurements

RT4 cells stably transfected with a NFκB luciferase reporter^62^ and maintained under G418 selection (Sigma, UK) at 0.5 mg/ml were seeded onto 30 mm diameter, six well plates at a density of 10^5^ cells/well and cultured until 90% confluent. Following challenge with either PBS, bacteria or bacterial components, the cells were lysed in RLB (Promega, Southampton, UK) for 16 hours at −80 °C and following the addition of luciferin (Promega), luminescence was measured using a FluoStar Omega microplate reader (BMG Labtech, Ortenberg, Germany). The results were presented as fold increase over the control PBS challenge.

Activation of TLR-5 was inhibited by incubating cells with up to 5 μg/ml of mouse monoclonal TLR-5 antibody (maba2-htlr5, Invivogen, San Diego, USA) or IgG (5 μg/ml) 2 hours prior to bacterial or flagellin challenge.

### End-point, Quantitative RT-PCR and siRNA analyses

Extraction of RNA from either cultured cells or the human tissue samples was performed using TRIzol® reagent following the manufacturer’s instructions (Invitrogen) and all RNA samples were stored in RNAsin^TM^ (Promega) at −80 °C. cDNA preparation and transcript abundance, measured by either end-point or quantitative RT-PCR, was as previously described^61^. Quality of extracted RNA was assessed using a Bioanalyzer 2100 (Agilent Technologies, Berkshire, UK) to ensure a minimum RIN of 8.0 for quantitative real time PCR (RT-qPCR) analyses of the *in vitro* samples and 7.0 for biopsy samples. Primers and annealing temperatures are presented in Table 1.

**Table 1:** Primer sequences.

Gene	Primer Sequence	Primer Type	Tm (°C)
*DEFB1*	Reverse: cgccGGTAGGAAGTTCTCATGGcG	Probes Master	60
	Forward: GTCAGCTCAGCCTCCAAAGGA		
*Defb4*	Forward: GTGAAGCTCCCAGCCATCAG	SYBR Green I Master	58
	Reverse: GATTGCGTATCTTTGGACACC		
*DEFB4*	SA Biosciences commercial primer	SYBR Green I Master	60
*DEFA5*	Forward: GCCATCCTTGCTGCCATTC	SYBR Green I Master	58
	Reverse: GATTTCACACACCCCGGAGA		
*hCAP-18*	Forward: cgctGACGGGCTGGTGAAGcG	Probes Master	55
	Reverse: CCCAGCAGGGCAAATCTCTT		
*TLR5*	Forward: CAGAGACTGGTGTTCAAGGAC	SYBR Green I Master	54
	Reverse: GTGTCCAGGTGTTTGAGCA		
*GAPDH*	Primer Design commercial primer	SYBR Green I Master	60
	(House-keeping reference gene)		
*ATP5B*	Primer Design commercial primer	SYBR Green I Master	60
	(House-keeping reference gene)		
Mouse β-actin	Forward: TGAGAGGGAAATCGTGCGTGACAT	SYBR Green I Master	60
	Reverse: ACCGCTCATTGACGATAGTGATGA		

Transfection of VK2 cells with TLR5 siRNA (s14199:ThermoFisher) was performed using Viromer Green (Lipocalyx) following the manufacturer’s protocol. The 48 h knockdown was followed by a 24 h flagellin challenge.

### Bacterial Growth Measurements

The immunosorbent experiments utilised urines collected from both flagellin and CFT073 challenged C57BL/6 wild-type mice. Urine samples were added to BD2 or Avian BD9 antibody (Cambridge Research Biochemicals, Cleveland, U.K.) coated ELISA plates (Leinco Technologies) and following a 2 h incubation the supernatants recovered and stored at −20 °C. Supernatants (100 µl/well) were used in bacterial growth experiments performed in 96 well microtitre plates and data analysed in relation to lag time extension^63^. For bacterial growth, over-night cultures of CFT073 were inoculated into Luria broth, grown to mid-log phase (OD_600_ = 0.4), diluted to 10^4^ cells/ml and aliquoted into wells of 96-well microtitre plates containing control or biological samples. The plates were incubated for 16 h in a Fluostar Omega plate reader set at 37 °C, shaken at 200 rpm every 20 min and the bacterial OD_600_ measured every 20 min.

### Human Beta-Defensin 2 (BD2) and Interleukin 8 (IL-8) Measurements

Human BD2 was quantitated in clinical and cell culture samples using a Human BD2 ELISA Development Kit (Leinco Technologies, St Louis, Missouri, USA). IL-8 concentrations were analysed using the BD optEIA IL-8 ELISA kit (BD Bioscience, Franklin Lakes, USA).

### Mice Experiments

C57BL/6J wild-type and B6.129S1-*Tlr5* ^*tm/Flv*^ */J (Tlr5* ^−/−^ knockout) female mice (obtained from Jackson Laboratories, Bar Harbor, ME.) were bred to between 6 and 8 weeks of age at the Duke University Medical Center animal care facility. *E*. *coli* CFT073 and *E*.*coli* flagellin were used for all murine infections. CFT073 cultures were inoculated from frozen stock into Luria broth [Becton Dickinson and Company (BD), Franklin Lakes, NJ] and grown overnight at 37 °C. Optical density (OD) was determined and cultures were washed, and diluted in PBS. Bladder infections were performed as described previously^6^. Essentially mice were anesthetized, catheterized with polyethylene tubing (inner diameter: 0.28 mm) (BD), and 50 μl of PBS (control) or PBS containing either 10^8^ bacteria or 5 μg *E*.*coli* flagellin instilled from a 1 ml tuberculin syringe with a 30G1/2 needle. Mice were euthanized by CO_2_ asphyxiation and whole tissues (vaginas, bladders and kidneys) isolated for transcript analyses and bacterial CFU quantification. All animal experiments were approved by the Duke University IACUC and Division of Laboratory Animal Resources, and performed in accordance with relevant guidelines and regulations.

### Human Subjects

The study was approved by the County Durham & Tees Valley 1 NHS Research Ethics Committee (09/H0905/15) and Newcastle upon Tyne Hospitals NHS Trust (ID 4841). Written informed consent was obtained from all participants and all methods were performed in accordance with relevant guidelines and regulations. Women with structurally-normal urinary tracts and consenting to provide biopsies, samples of blood, urine and vaginal washings were recruited from the Urology and Uro-gynaecology departments of Newcastle upon Tyne Hospitals NHS Trust from July 2009 to February 2011. Controls were recruited from women attending for investigation of haematuria or other non-infection related uro-gynaecological assessment; rUTI sufferers were women who had suffered either at least two episodes per year for two years or three episodes in the previous year. Other inclusion criteria required subjects to be aged 18 years or older; pre-menopausal or at least six months post−menopause; no antibiotic therapy within four weeks of recruitment. Pre-investigation sample size calculations based on previous reported urinary AMP concentrations indicated that 18 women in each of the four groups (72 in total) would give 80% chance of detecting a difference at 5% significance level. The women were stratified according to menopausal status to create four groups: 19 Pre-menopausal controls (median age 35: range 18–46); 31 Pre-menopausal cases (rUTI sufferers) (median age 31: range 18–41) for which there were 26 complete analyses; 19 Post-menopausal controls (median age 58: range 42–76); 29 Post-menopausal cases (rUTI sufferers) (median age 60: range 40–75) for which there were 22 complete analyses. Clinical histories of rUTI and control subjects are presented in Table 2.

**Table 2:** Clinical History.

	Control		rUTI	
	Pre-menopause	Post-menopause	Pre-menopause	Post-menopause
Numbers Recruited	19	19	31	29
Median Age (Range)	35 (18–46)	58 (42–76)	31 (18–41)	60(40–75)
Prescribed vaginal estrogen previously	1	2	0	0
Taking vitamins	4	3	4	4
Taking cranberry-juice	5	5	16	15
On oral contraception	6	0	4	0
Had HRT	0	4	0	9

### Patient samples

Biopsies from the first 2–3 cm of the vagina and posterior bladder wall were obtained during cystoscopy. Following cystoscopy, the first vaginal washing or douche was collected using the ‘Summer’s Eve Cleansing Douche’ (Fleet Laboratories, Lynchburg, Virginia, USA) according to the manufacturer’s instructions. Each subject was given the necessary instructions and containers to collect an overnight urine specimen (approximately 600–800 ml) and a further vaginal douche 6–8 weeks later. Pre−menopausal subjects were requested to collect the douche at the mid−point of their menstrual cycle. Of the subjects that gave two douche samples, all were post-menopausal; nine were rUTI sufferers prescribed vaginal estrogen (Vagifem twice weekly 10 mcg) and 12 were rUTI sufferers given treatments including antibiotic prophylaxis and/or advice (one did not complete).

### Targeted TLR5 Sequencing

Blood samples taken from each subject were processed for genomic DNA (gDNA) extraction using the GeneCatcher™ gDNA 3–10 mL Blood Kit (Invitrogen, Paisley, UK). PCR amplification of the TLR5^392S*top*^ containing region of the TLR5 gene was carried out on 100 ng of gDNA using Novagen Kod Host start DNA Polymerase (Merck Millipore, Darmstadt, Germany) and the TLR5 Primer pair (forward primer, GGTAGCCTACATTGATTTGC; reverse primer, GAGAATCTGGAGATGAGGTACCCG). A 10 μL aliquot of the 461 bp product was used for sequencing (Genevision, Newcastle upon Tyne, UK). Sequencing data was provided in AB1 format and data analyses performed using the freeware FinchTV trace viewing software (Geospiza, Seattle, USA).

### Immunohistochemistry

Vaginal samples for immunohistochemistry were fixed in formalin overnight before being transferred to 70% ethanol for long term storage or paraffin embedding. Sections of 5-µm thickness were mounted on microscope slides and following microwave-heat mediated antigen retrieval were stained with anti-beta 2 Defensin primary antibody (Abcam, ab63982)1/1000 (1 X PBS), overnight at 4 °C, followed by one hour at room temperature with polyclonal goat anti-rabbit Immunoglobulins/HRP 1/3000 (DAKO). Images were taken using a Nikon Eclipse Ti, coupled to a Photometrics Coolsnap HQ CCD camera and Nikon Plan Fluor 100x/1.30 ph3 DL lens.

### Statistical Analyses

All statistical analyses were performed out using the Prism 5 Software package (GraphPad Software Inc, La Jolla, California, USA). Significance of data with two groups was determined by unpaired two-tailed Student’s t test; for more than two groups a two–way analyses of variance followed by Tukey’s multiple comparison or Bonferroni post-tests, as appropriate, was used. For comparison of the first and second douche data, a paired two-tailed test was utilised.

## Electronic supplementary material

Supplementary Data
