# Supplementary material for: Targeting Deficiencies in the TLR5 Mediated Vaginal Response to Treat Female Recurrent Urinary Tract Infection
Source: Sci Rep. 2017 Sep 8;7:11039. doi: 10.1038/s41598-017-10445-4 (PMC5591273; doi:10.1038/s41598-017-10445-4)

Targeting Deficiencies in the TLR5 Mediated Vaginal Response to Treat Female Recurrent Urinary Tract Infection.

Authors: Ased SM Ali[1,2a], Catherine Mowbray[1], Marcelo Lanz[1], Anna Stanton[1], Samantha Bowen[3], Claire L Varley[4], Paul Hilton[2], Karen Brown[2], Wendy Robson[2], Jennifer Southgate[4], Phillip Aldridge[1], Alison Tyson-Capper[1], Soman Abraham[3], *Robert S Pickard [1,2*], Judith Hall[1*],

*Judith Hall and Robert S Pickard contributed equally to this work as senior supervising authors.

Institutions:

1. Institutes of Cell & Molecular Biosciences and Cellular Medicine, Newcastle University, UK
2. Newcastle upon Tyne Hospitals NHS Trust, UK
3. Duke University, NC, USA
4. Jack Birch Unit, Department Biology, University of York, UK

[a] Present address Mid Yorkshire Hospitals, Aberford Rd, Wakefield, UK

Corresponding Author:

Dr Judith Hall, Institute for Cell & Molecular Biosciences, Faculty Medical Sciences, Newcastle University. Telephone: +44 (0) 1912088346. Email: Judith.Hall@ncl.ac.uk

Supplementary Information

Supplementary Data

Figure S1: Full-length gel showing host defence peptide gene expression in VK2 E6/E7 following 24hr challenge with PBS (control) **S1a;** Full length gel showing host defence peptide gene expression in VK2 E6/E7 following 24hr challenge with flagellin (250ng/ml) **S1b;** Full length gel showing host defence peptide gene expression in VK2 E6/E7 cells following 24hr challenge with UPEC (10^5) **S1c;** Full length gel showing host defence peptide gene expression in RT4 cells following 24hr challenge with PBS (control) or flagellin (250ng/ml). Marker/M (DNA ladder) **S1d.**

S1a: VK2 control

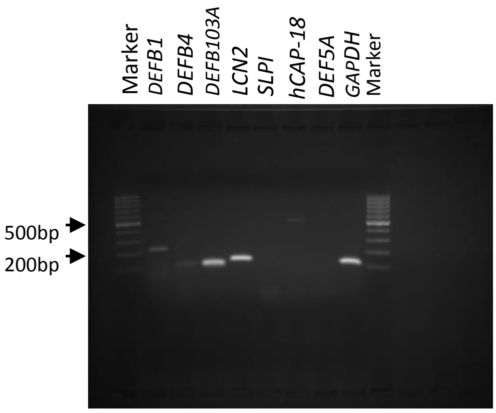

S1b: VK2 flagellin

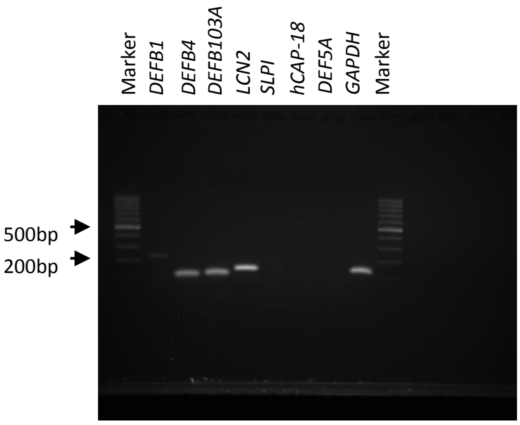

S1c: VK2 UPEC

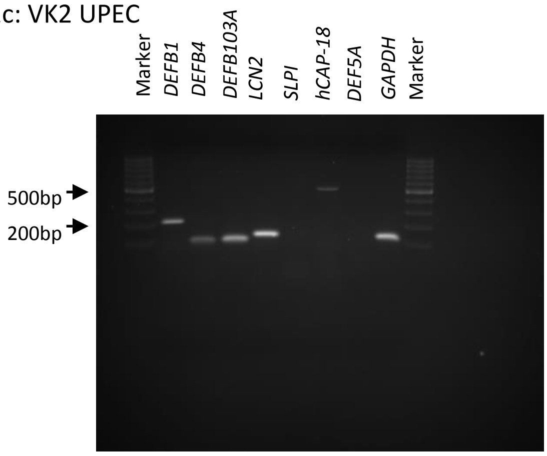

S1d: RT4 control and flagellin challenges

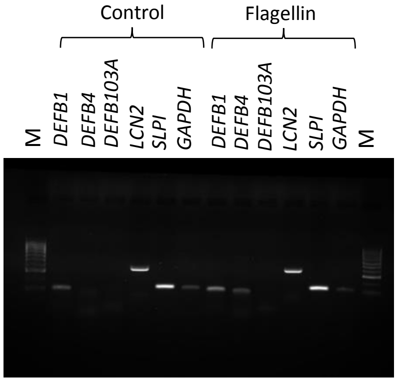

Figure S2: *DEFB1* transcript expression, presented as relative expression, of RT4 cells **(a)** and VK2 E6/E7 cells **(b)** challenged with either PBS, 10^5 killed NCTC 10418 (highly flagellated) or NU14 (non-motile) for up to 24 hours (N=3; n=9).

a) RT4 b) VK2 E6/E7

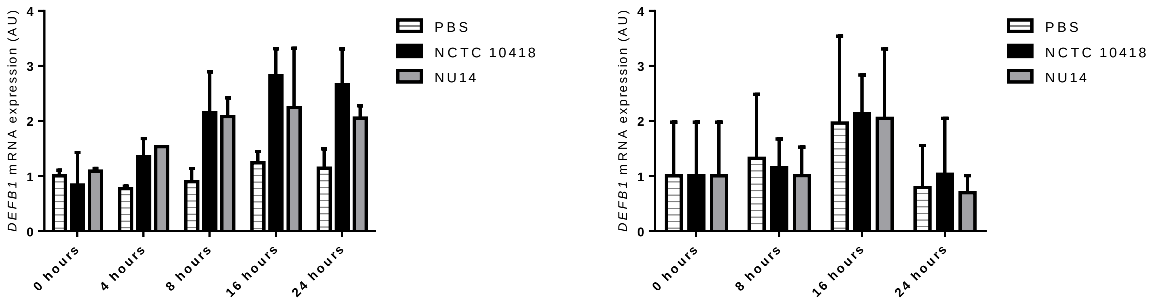

Figure S3: *DEFB4* transcript expression, presented as relative expression, of RT4 cells **(a)** and VK2 E6/E7 cells **(b)** challenged with either PBS or LPS (10μg/ml) for up to 24 hours (N=3; n=9).

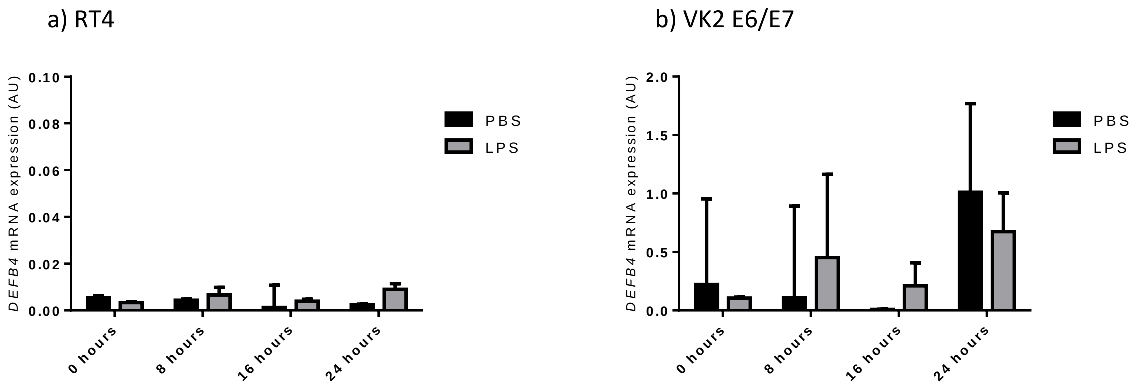

Figure S4: BD2 concentrations (pg/ml) in media of VK2 E6/E7 cells incubated in presence or absence of TLR5 blocking antibody (5ug/ml) and challenged for 24 hours with either flagellin (250ng/ml) or bacteria isolated from each of the three SNP patients (N=2; n=6).

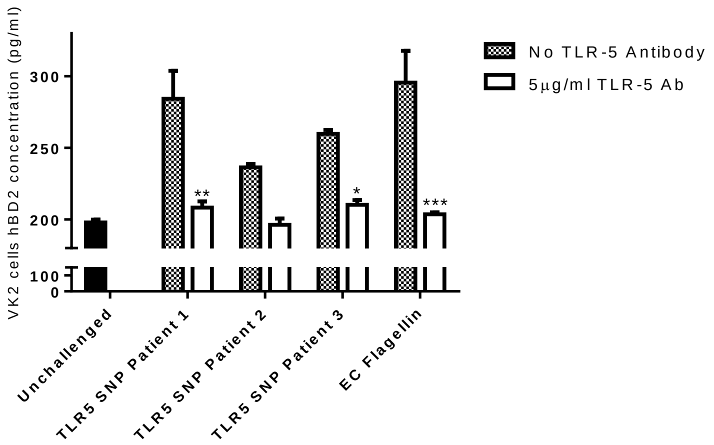

Supplement: Supplementary file 1 — Supplementary Data [file 41598_2017_10445_MOESM1_ESM.pdf]
